# Impact of acute mental fatigue on cricket-related performance measures in university level indoor cricket players

**DOI:** 10.3389/fspor.2025.1527696

**Published:** 2025-05-30

**Authors:** Michelle Evans, Travis Blood, Rachel L. Bevins

**Affiliations:** ^1^ School of Health and Rehabilitation Sciences, Health Sciences University, Bournemouth, United Kingdom; ^2^ School of Science, Coventry University, Coventry, United Kingdom

**Keywords:** cricket, mental fatigue, athlete monitoring, sport performance, team sports

## Abstract

Acute mental fatigue affects elements of sporting performance such as technical performance or decision making in high-level athletes, however less is known about the effects in non-elite sport. The aim of this study was to examine the effect of acute mental fatigue on cricket specific performance and reaction time in university-level cricketers during the indoor competitive season. Ten male university cricket players (Age 18–23 years, height 183.3 ± 8.7 cm, body mass 88.5 ± 19.8 kg) performed baseline testing, and two experimental test conditions separated by a 48-hour washout. Mental fatigue was induced using two different tasks: a 30-min smartphone-based Stroop test (Stroop) and a 30-min smartphone-streaming based cricket video (Film). Performance outcomes were assessed through the English Cricket Board's Run2 test for sprint performance and the Batak test for reaction time. The results indicated that both the Stroop and Film conditions induced mental fatigue compared to Baseline [χ^2^ (2) = 19.16, *p* < 0.001], although the Film condition produced only a small increase in fatigue. Cricket Run2 times were negatively affected by both the Stroop and Film tasks [F(2) = 24.83, *p* < 0.001]. Acute mental fatigue, induced by either an app-based Stroop test or an app-based video stream, negatively affected cricket-relevant performance in university level indoor cricketers.

## Introduction

Cricket involves prolonged low-intensity activity interspersed with repeated high-intensity bouts (1, 2). Cricket has a variety of outdoor formats such as multi-day, limited overs and shorter versions like Twenty20, each requiring different intensities. Indoor cricket, with fewer players and less overs per side, is typically higher in intensity (1). Beyond its physical demands, cricket poses significant perceptual and cognitive challenges (3), especially in tasks batting (4) and fielding (2), where quick and accurate decisions are key. Prolonged batting (>2 h) affects cognitive performance in youth players, particularly in higher order tasks (5), whereas mental fatigue impairs cricket running performance in professionals (6).

Mental fatigue, which arises from prolonged cognitively demanding activities, is characterised by feelings of tiredness, reduces cognitive performance (7) and exercise tolerance (8), negatively influences manual dexterity and coincidence anticipation (9), and increases reaction times to visual stimuli (10). Team sports are cognitively demanding due to their unpredictable nature (11) and in soccer, it impairs both physical and technical performance (12), including performance in small-sided games (13). Similar effects appear across other sports such as running performance in cricket (6) and handball (14), running performance and goalkicking in Australian rules football (15), swimming performance in young athletes (16), and basketball shooting performance (17). In professional female volleyball players, fatigue impairs visual perception, concentration, and reaction time, showing its broad impact (18). Cognitive functioning underpins sports performance, influencing decision making and reaction speed (19). Understanding cognitive fatigue is vital in mentally demanding sports, as it may compromise crucial skills such as anticipation and motor control. Most research has focussed on elite athletes (20), who may be more resistant to mental fatigue through superior inhibitory control (21, 22). Therefore, research into non-elite athletes who may train less and may have fewer resources to manage fatigue, is warranted.

Recent studies have begun exploring how smartphone apps (23) or video gaming (24) affect mental fatigue and sporting performance. Athletes often use social media, games and video streaming prior to and, potentially for cricketers, during competition (for the batting side whilst not in the field of play). In both soccer and boxing, 30 min of social media use or gaming impaired decision-making performance, with soccer players showing reduced passing decision making performance (23) and boxers demonstrating poorer attack and defence decisions compared to a control video (24). Both these studies used a video condition as the control, however Daub et al. (17) found in basketball players that watching sports-specific film did result in an increase in mental effort and a large though not significant increase in mental fatigue.

Mental fatigue in cricket may vary across formats, from short games to longer Test matches (6). However, research on mental fatigue in shorter formats such as indoor matches is limited which require all players to bat and bowl, increasing cognitive and physical demands. This study aims to examine the acute effects of a mentally fatiguing smartphone task vs. watching a cricket video on sprint and reaction performance in University-level indoor cricketers. It was hypothesised that both conditions would induce mental fatigue due to smartphone use, negatively affecting sprint and reaction performance.

## Material and methods

### Participants

Ten male University cricket players (age range 18–23 years, height 183.3 ± 8.7 cm, body mass 88.5 ± 19.8 kg) currently competing in the indoor cricket season volunteered to participate in this study. All participants had at least two years of experience playing cricket at any competitive level but were exclusively recruited from the current University first team. None of the participants had visual impairments or were colour blind. They were instructed to follow their regular diet the day before testing and to maintain the same diet consistently across all three testing sessions. Participants were provided with verbal and written instructions outlining the study procedures and provided written informed consent. Institutional ethical approval at Department level (ethics: P143914) was provided for this study, which was conducted in accordance with the Declaration of Helsinki.

### Study design

**Figure 1:**
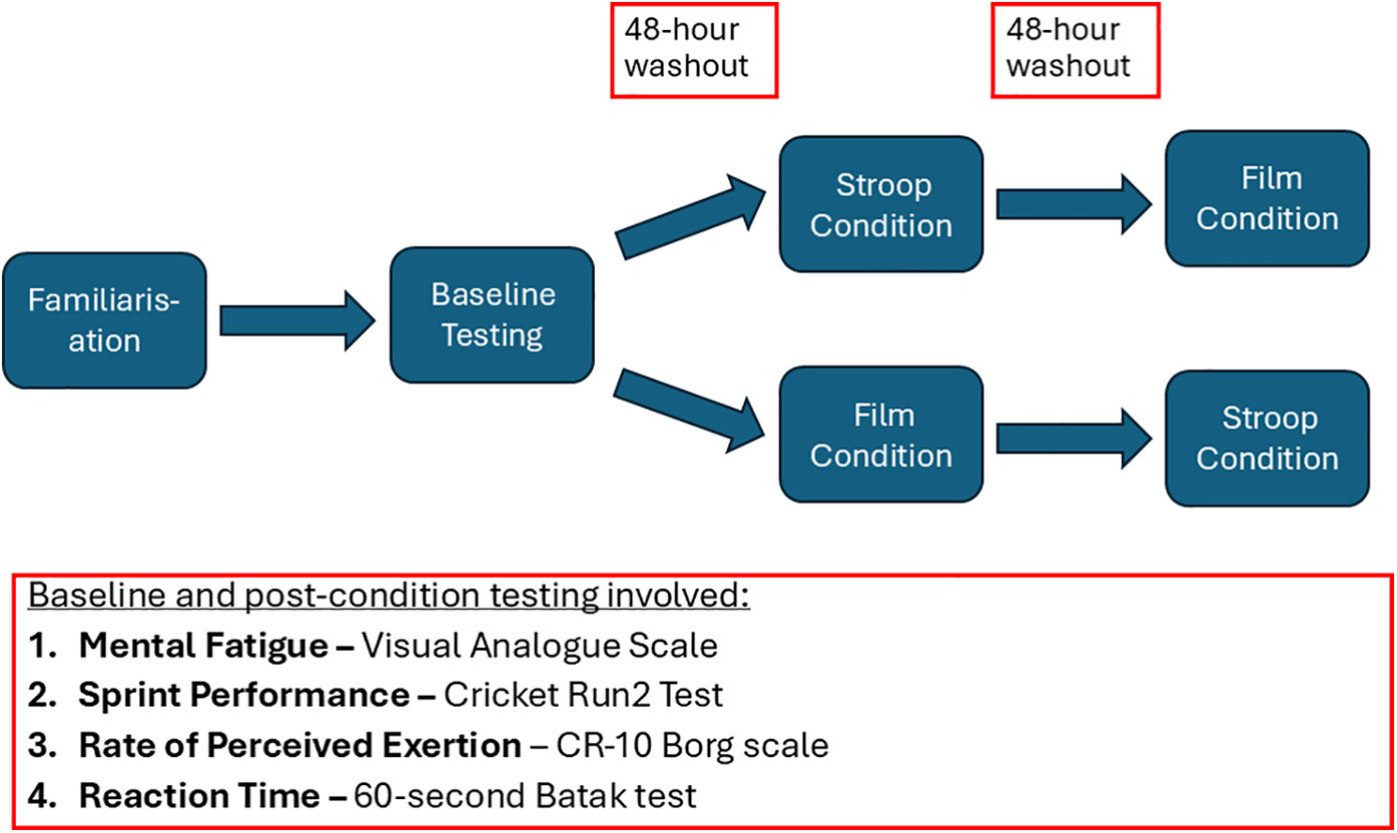
Schematic timeline of study design.

Participants visited the testing facility on four separate occasions, with the first visit functioning as a familiarisation session for the testing procedures. The second visit functioned as the baseline assessment (Baseline) where the performance outcome measures were conducted in the absence of a mentally fatiguing treatment condition. The third and fourth visits comprised of two experimental sessions (Film and Stroop), which were performed in a counter-balanced order to reduce carry-over effects (see Figure 1). The procedures in each session were the same, with the only difference being the intervention conducted prior to the testing procedures. There was a 48-hour washout period between each condition (17).

### Fatigue inducing regimens

To induce mental fatigue, participants completed a 30-min app-based Stroop test on an iPhone [Brain Test - Stroop Effect, App Store (https://www.stroopeffectapp.com)]. This was a 60-s protocol conducted in “Classic” mode which was played continuously for the 30-min condition duration. The participants were encouraged to answer as accurately and quickly as possible throughout the entire 30-min testing period. A 30-min Stroop task has previously been shown to increase mental fatigue without affecting motivation (6, 17).

In addition to the Stroop condition, participants also took part in a 30-min Film condition, during which they watched a cricket-specific video review (highlights from the England vs. New Zealand One-Day Internationals 2014). The video was viewed on an iPhone via the online platform YouTube (San Bruno, California, United States).

### Outcome measures

After each treatment condition, participants completed the following measures in the same order: the mental fatigue scale, sprint performance, and a 60-s Batak test.

Participants subjectively rated their level of mental fatigue using a visual analogue scale (VAS) (25). The scale was anchored with the words “none at all” at the 0-score and “maximal” at the 10-score.

Sprint performance was measured using the English Cricket Board Run2 test. This test required the participant to run a distance of 17.68 m (the distance between the two popping creases on a cricket pitch) with a standard cricket bat in their preferred hand (6, 26). They were required to touch the bat down over the crease, turn 180°, and run back to the starting point, simulating the action of scoring two runs in a cricket match (6). Infrared timing gates (Brower Timing T-Ci System, Brower Timing Systems, Utah, USA) were positioned at the start of the 17.68 m line at a height of 1 m, and a time was recorded for each Run2 test where participants repeated the test three times with the average score recorded for analysis. Participants wore appropriate clothing and footwear for sports testing but did not wear cricket-specific protective gear such as helmet, gloves, and pads. The CR-10 Borg scale (27) was used after each condition to assess rate of perceived exertion (RPE) after the sprint testing.

For reaction time, participants completed the 60-s Batak (Batak Micro, Quotronics Limited, UK) accumulator programme to measure their reaction scores. The portable Batak system (600 m × 600 m × 70 mm) features 12 Polycarbonate translucent light-emitting diode (LED) targets, with participants earning a point for each target pressed. The total points were recorded after each test, and the average score from three trials was used as the final score. Participants performed the test in a crouched position, with the Batak system placed at hip height on a table to simulate fielding positions in cricket.

### Statistical analysis

All data are presented as means ± standard deviations (SD) unless otherwise stated. Data for the Run2 and reaction time tasks were verified for normality using the Shapiro–Wilk test, and for sphericity using Mauchly's test. Differences between conditions for these tasks were then investigated using repeated measures ANOVA. The location of any significant differences were then investigated with *post hoc* analyses using the Bonferroni correction. Differences between conditions in VAS and RPE scores were investigated using Friedman's test. If significant differences were detected, the location of these was investigated through *post hoc* analysis using the Wilcoxon Signed rank test. The level of statistical significance was set at *p* ≤ 0.05.

## Results

**Figure 2:**
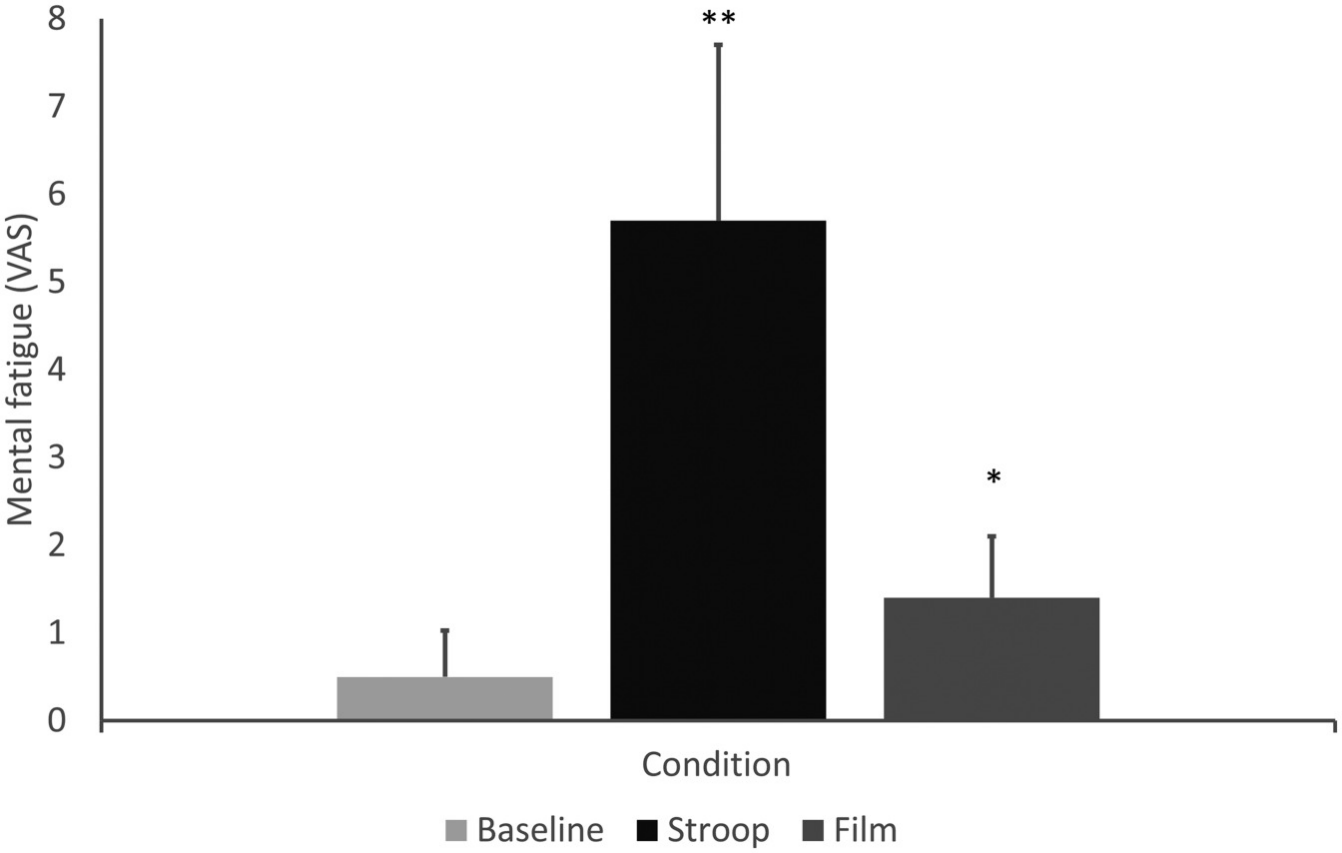
Perceived level of mental fatigue at baseline, and after the stroop and film conditions; *indicates a significant difference from baseline, **indicates a significant difference from baseline and film conditions.

Mental fatigue assessed through VAS showed a significant difference between groups [χ^2^ (2) = 19.16, *p* < 0.001; Figure 2]. *post hoc* tests using Wilcoxon Signed rank test showed the Stroop (VAS 5.7 ± 2.0) condition to elicit higher levels of mental fatigue than both the Baseline (VAS 0.5 ± 0.5; *Z* = −2.81, *p* = 0.005) and Film (VAS 1.4 ± 0.7); *Z* = −2.82, *p* = 0.005) conditions. The Film condition also elicited higher mental fatigue than the Baseline condition (*Z* = −2.71, *p* = 0.007).

**Figure 3:**
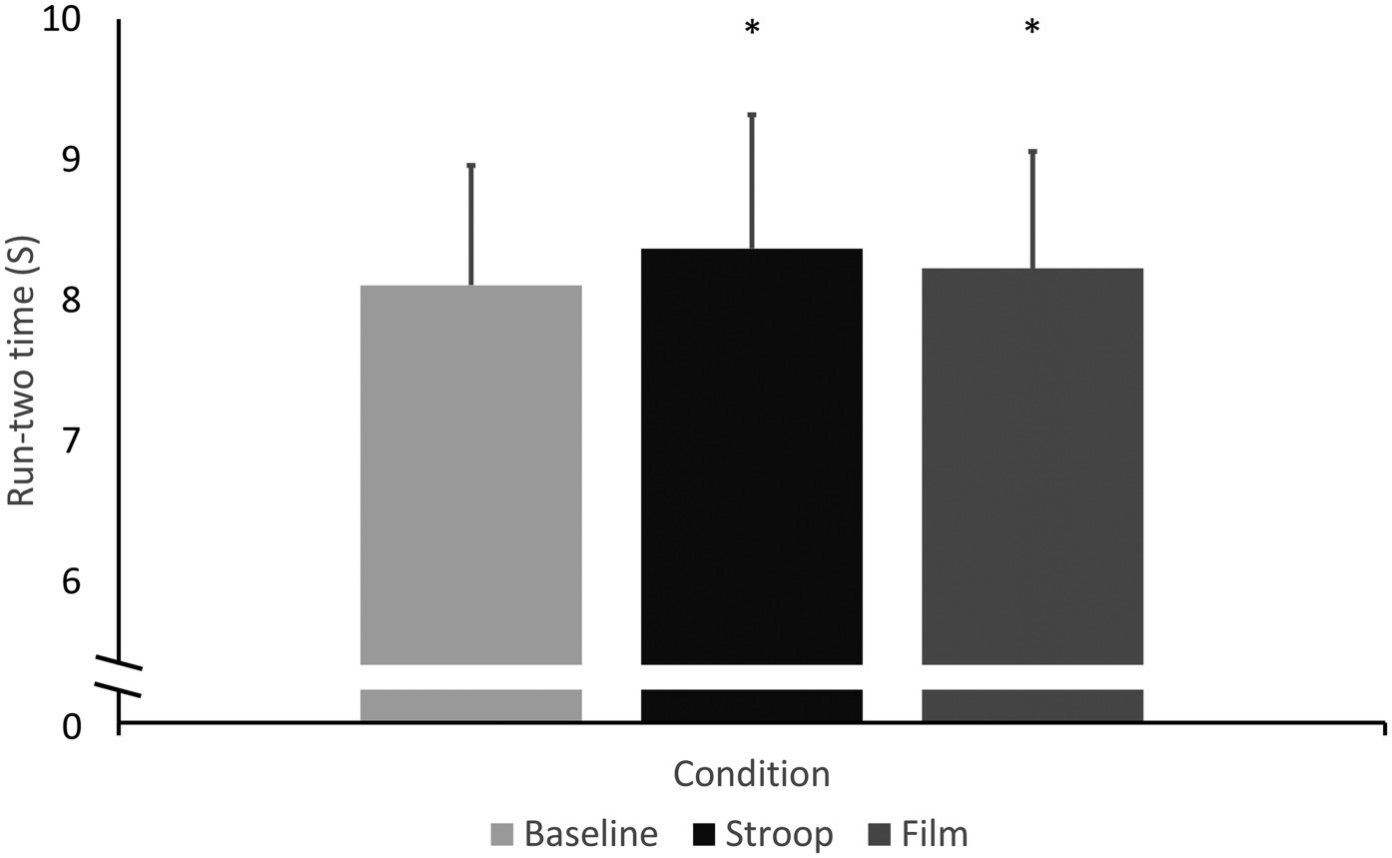
Cricket run2 times at baseline, and after the stroop and film conditions; *indicates a significant difference from baseline.

Repeated measures ANOVA revealed a significant difference between groups for the Run2 times [F(2) = 24.83, *p* < 0.001, partial eta squared = 0.861; Figure 3]. *post hoc* analysis using a Bonferroni correction revealed that both the Stroop condition (8.37 ± 0.86 s; *p* < 0.001) and the Film condition (8.2 ± 8.3 s; *p* = 0.018) were significantly slower than the Baseline condition (8.11 ± 0.86 s). The difference between times was not significant between the Stroop and Film conditions (*p* = 0.234).

There were no significant differences between groups (Baseline 118.0 ± 9.4; Stroop 116.3 ± 9.4; Film 119.8 ± 11.7) for reaction time assessed with the Batak system [F(2) = 1.00, *p* = 0.386, partial eta squared = 0.100]. No significant differences were detected between groups (Baseline 2.1 ± 1.3, Stroop 1.6 ± 0.9, Film 2.4 ± 1.2) for RPE [χ^2^ (2) = 3.94, *p* = 0.139].

## Discussion

The primary aim of this study was to examine the effects of acute mental fatigue, induced by the Stroop task on a smartphone-based app (Stroop) and by a cricket video task (Film), on cricket related sprint performance and reaction time in university level cricket players involved in the Indoor competitive cricket season The key findings showed that both the Stroop and Film conditions induced mental fatigue compared to baseline, though the Film condition caused only a slight increase in fatigue. This mental fatigue resulted in a decrease in performance on the cricket Run2 test, but no significant change in reaction time assessed with the Batak system.

The perception of acute mental fatigue was successfully induced by the Stroop test in this study and was comparable to the mental fatigue produced by a previous study using cricketers (6). The Stroop test has been widely used in previous research investigating the effects of mental fatigue on aspects of sporting performance (20). There has been some criticism that the Stroop test has low ecological value when considering mental fatigue induced by sport (28), however this test has been demonstrated to effectively induce mental fatigue (6, 17) and is the most commonly used cognitive task utilised in studies of this nature (11, 29).

An increase in perception of mental fatigue was also induced by the Film condition, though this did not reach the suggested cut-off value of 3.5 (30). Both of these conditions used a mobile phone as the platform. Previous research has also found an increase in mental fatigue as a result of documentary film watching on a smartphone and found this to be similar to social media use (31). The higher level of fatigue induced by the Stroop condition, indicates that when interacting with a game-based app rather than just watching videos on mobile phones leads to a higher level of fatigue. However, both Stroop and Film conditions had similar deleterious effects on the Run2 performance measure, despite the Film condition not producing as high a perception of mental fatigue. This is an important finding, because there is increasing use of social media and video streaming pre-match in sport (32), and it is likely that cricket players will utilise phone apps and games both pre-match and potentially in the amateur game during the match whilst waiting to bat after their innings.

The Run2 times in this study (Baseline 8.10 ± 0.86 s) were slower than those previously reported (6, 26), however as this study used recreational level players rather than elite professional players this finding is unsurprising. When the mean change in times is viewed for the elite players the Stroop condition resulted in a 0.10 s slower time to complete the test (6), whereas in the current study the Film condition and the Stroop condition resulted in a 0.13 s and 0.26 s slower time respectively. Although these changes sound small, these performance differences may have large competition impacts (16, 22), and every single participant in this study had a slower Run2 time in the Film and Stroop conditions than in the Baseline condition.

Although the Run2 test sounds simple in nature, it requires the participants to control a cricket bat in one hand, whilst accelerating, running at maximal speed, decelerating to perform a 180° turn and then accelerating again to return to the starting position (6). Therefore, this test depicts several key skills involved in cricket, and as shorter formats of cricket are performed at higher intensity than multi-day formats (1) and run rate is a significant performance indicator in short format games (33), it is likely that the batsmen in indoor cricket are going to be repeatedly sprinting to maximise scoring potential. Mental fatigue is believed to negatively impair preparation and planning, and therefore can affect the ability to respond to cues for future decision making (20). In a cricket context, this could be reflected in appropriate shot selection based on the ball delivered, or judging whether to run a quick single or whether there is scope to turn a single into two runs.

There was a lack of significance between conditions for the Batak scores, replicating previous findings that used elite players (6). Although this study positioned the Batak system at a height to try and replicate responding to the ball, e.g., close catch position such as fielding in the slip cordon, the test may not be sports specific enough to truly assess the impact of acute mental fatigue on elements of cricket performance (34). Mental fatigue does affect both manual dexterity and anticipation timing (9), resulting in increased reaction times and decreased accuracy (35), therefore it is reasonable to assume that despite no decline being detected on tests using the Batak, as a more complex and/or cricket specific task such as ball tracking or shot selection, may have greater sensitivity for detecting decreases in performance (34, 36).

Future research could include outcome measures that closely replicate the intermittent demands of batting, bowling and fielding in cricket across the different game formats. For example the use of global positioning systems (GPS) may provide data on movement patterns in batting and fielding that could detect any sports-specific decrements in performance. Whether watching cricket itself is mentally fatiguing, i.e., following the game that is in play whilst waiting to bat, or whether it is the nature of watching on a screen that is subsequently detrimental to performance is beyond the scope of this study, but these effects could be further elucidated in future protocol designs.

As mental fatigue has a negative effect on cricket performance in both elite (6) and non-elite players, future research could evaluate the utility of strategies to reduce mental fatigue. For example, within padel, performance in both rested and a sleep-deprived state were improved when the physical warm-up was interspersed with short cognitive tasks compared to a physical-only warm-up (37). Caffeine ingestion has shown efficacy both during and after a mentally fatiguing task (38), however caffeine does have potential undesirable side effects such as gastrointestinal distress. Research on interventions such as mindfulness or relaxing music is promising but has only been conducted in a limited number of sports and has only considered technical outcomes (39).

In conclusion, cricket-related run performance is negatively affected by mental fatigue in non-elite players involved in a short-format indoor version of cricket. These findings are concordant with previous work (6), and extend the findings from elite to non-elite players in a different game format. The results also discovered that previously reported mental fatigue induced by smartphone use (31) resulted in a comparative reduction in run performance as the Stroop test. Therefore, the outcomes of this study suggest that University level Indoor cricketers should abstain from cognitively demanding phone apps and games as well as video streaming prior to training practice and matches.

## Data Availability

The original contributions presented in the study are included in the article/Supplementary Material, further inquiries can be directed to the corresponding author.
